# Nonsteroidal ^18^F-Labeled PET Tracer for Imaging Androgen Receptors

**DOI:** 10.2967/jnumed.125.271531

**Published:** 2026-09

**Authors:** Vilma I.J. Jallinoja, Joo Sun Mun, Alexandra Campanella, Aahna Sasson, Teja M. Kalidindi, Giovanna Caxeiro, Darren R. Veach, Serge Lyashchenko, Heiko Schöder, Naga Vara Kishore Pillarsetty

**Affiliations:** 1 Department of Radiology, Memorial Sloan Kettering Cancer Center, New York, New York; and; 2 Department of Radiology, Weill Cornell Medicine, New York, New York

**Keywords:** androgen receptor, prostate cancer, imaging, PET, ^18^F

## Abstract

Androgen receptor (AR) signaling is the key driver of prostate cancer. Thus, standard-of-care treatments focus on androgen-deprivation therapy followed by AR signaling inhibitors. Even when initially responsive, patients eventually develop resistance to therapies targeting AR signaling, leading to disease progression. To study lesion-to-lesion AR occupancy, the radiofluorinated analog of the endogenous androgen [^18^F]16β-fluoro-5α-dihydrotestosterone ([^18^F]FDHT) has been investigated as a potential AR imaging agent. In the literature, [^18^F]FDHT demonstrates slow clearance from healthy tissue, poor plasma stability in vivo, and nonapplicability in mouse studies. To overcome these limitations, we explored nonsteroidal selective androgen receptor modulator (SARM)–based pharmacophores as potential PET tracers. Here, we describe the performance of a ^18^F-radiolabeled AR tracer, [^18^F]F-SARM3. **Methods:** [^18^F]F-SARM3 was synthesized via 1-step copper-catalyzed radiofluorination, and its AR affinity (half-maximal inhibitory concentration [IC_50_]) and biological activity (half-maximal effective concentration [EC_50_]) were studied in various prostate cancer cell lines. The tracer’s stability in vitro was also evaluated. [^18^F]F-SARM3 was evaluated in LNCaP and 22Rv1 (AR-positive) tumor–bearing male mice to assess its AR specificity and clearance profile, and its performance was compared with that of [^18^F]FDHT. **Results:** [^18^F]F-SARM3 was synthesized with a radiochemical yield and purity of 2.7 ± 1.4% and 97.9 ± 2.3%, respectively. In in vitro cell-binding assays, [^18^F]F-SARM3 demonstrated high affinity for ARs, similar to that of dihydrotestosterone (IC_50_, 20.2 ± 14.6 nM vs. 9.6 ± 4.0 nM, respectively, in 22Rv1 cells). [^18^F]F-SARM3 functions as a partial AR agonist, with an EC_50_ of 15.0 ± 12.0 nM. Furthermore, [^18^F]F-SARM3 is highly stable in phosphate-buffered saline and mouse blood (98.5% ± 1.2% and 98.6% ± 1.5% intact, respectively). The tracer’s in vivo specificity was confirmed with the observed uptake in tumors and prostate glands of castrated mice, where uptake was blockable. In vivo specificity was not observed with [^18^F]FDHT. **Conclusion:** We developed a first-in-class SARM-based AR tracer that displays high affinity and selectivity for AR in vitro and in vivo. This represents a suitable PET tracer to image AR status in rodent models and provides a strong rationale for clinical translation of [^18^F]F-SARM3 as a high-affinity AR agonist PET imaging agent.

## Visual Abstract

**Figure:**
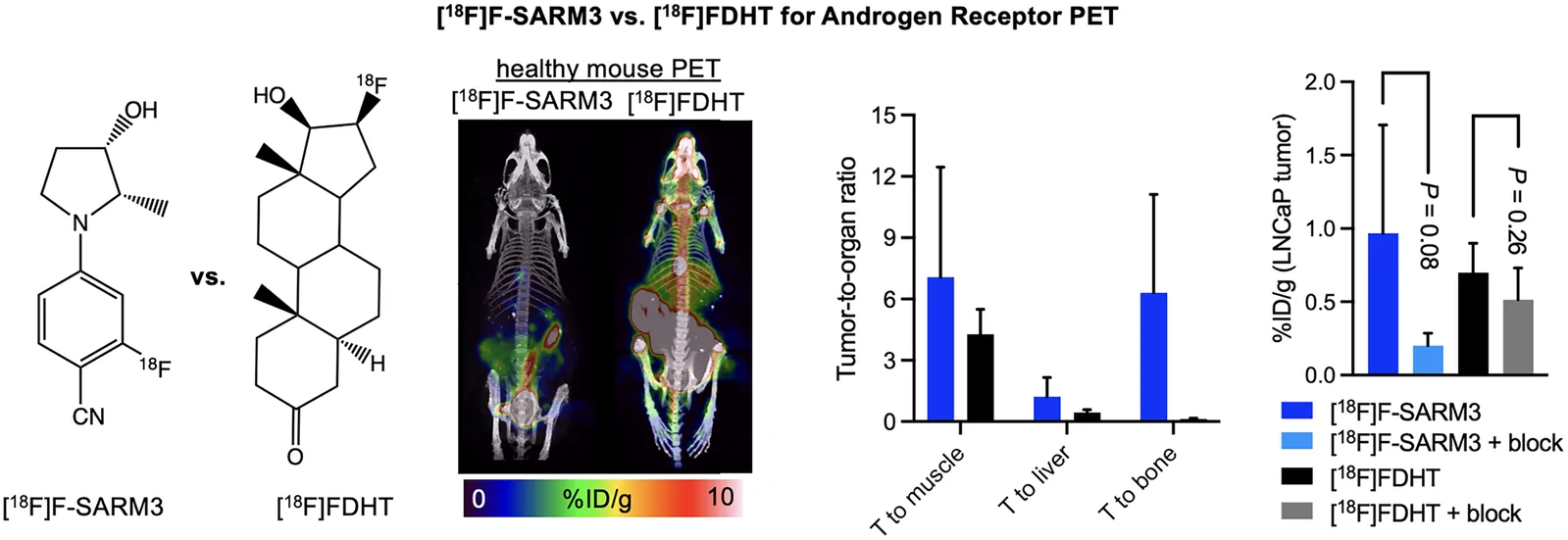

Androgen receptor (AR) signaling is the key driver in the development and progression of prostate cancer, the second deadliest cancer among men in the United States (1). From early-stage, hormone-sensitive, localized disease to metastatic castration-resistant prostate cancer, the standard-of-care treatment remains the inhibition of AR signaling via androgen-deprivation therapy or androgen receptor signaling inhibitors (e.g., enzalutamide) (2). Eventually, all patients develop resistance to these AR-targeted therapies via a variety of mechanisms, including AR amplification, AR mutations, or the complete loss of AR dependence, resulting in AR-null tumors that do not respond to AR signaling inhibitors (3). Thus, understanding the presence or absence of AR in prostate cancer lesions is essential to guide optimal treatment regimens. Only one AR PET tracer, [^18^F]16β-fluoro-5α-dihydrotestosterone ([^18^F]FDHT), has been studied in the clinical setting for its ability to quantify lesion-to-lesion AR heterogeneity (4–6). However, [^18^F]FDHT works only in patients with prostate cancer who have undergone castration and have low physiologic androgen levels. Although the natural binding of [^18^F]FDHT to sex hormone–binding globulin (SHBG) in humans does increase the tracer’s stability and extend its blood pool residence time, [^18^F]FDHT is rapidly metabolized and suffers from poor in vivo stability and high background signal because of slow blood clearance of tracer metabolites. The tracer is not suitable for imaging AR-positive tumors in mice models because of the complete lack of SHBG, resulting in rapid clearance of the intact [^18^F]FDHT from the blood pool and its rapid metabolism that leads to defluorination of the tracer (7–9). Moreover, its clinical use has been further limited by the technical difficulties reported in clinical [^18^F]FDHT production, including its complicated 3-step radiosynthesis (10). An additional significant but underappreciated challenge is that, because of the lack of a chromophore, specific activity measurements are impeded, potentially resulting in significant differences among batches, which can affect uptake in tumors.

To address these challenges, we developed a ^18^F-radiolabeled AR-targeting tracer, [^18^F]F-SARM3, using a 4-(pyrrolidin-1-yl) benzonitrile scaffold that functions as a selective androgen receptor modulator (SARM), as reported by Asano et al. (11). In their report, they described a series of 1-(4-(pyrrolidin-1-yl) benzonitrile-2,3-substituted analogs, revealing that the hydroxyl group at the 3-position on the pyrrolidine ring and the cyano group at the 4-position of the phenyl ring of the pharmacophore were essential for AR binding. Notably, substituents at the 2 and 3 positions on the phenyl ring did not appear to significantly influence AR binding. Guided by these findings, we designed our potential tracer compounds and investigated several fluorinated 4-(pyrrolidin-1-yl) benzonitrile derivatives. Among these, F-SARM3 was selected as the final candidate because of its superior AR binding affinity and relative ease of radiolabeling. We hypothesized that our non-SHBG–binding, high-affinity, high-stability SARM pharmacophore could lay the foundation for the development of an AR tracer. Here, we report the successful preclinical development and evaluation of [^18^F]F-SARM3 in a mouse model of prostate cancer and compare its in vivo performance with that of [^18^F]FDHT.

## MATERIALS AND METHODS

Details on the chemicals and instruments used are provided in the supplemental materials, available at http://jnm.snmjournals.org.

### Animal Models

All experiments were performed under Animal Protocol 13-06-005 of the Memorial Sloan Kettering Cancer Center Institutional Animal Care and Use Committee. Male outbred athymic nude mice (8 wk of age) from The Jackson Laboratory were used for all in vivo studies. For human tumor xenograft experiments in mice, the cells were injected subcutaneously into the right shoulder of the mouse (5 × 10^6^ LNCaP cells [passage, <10] or 2 × 10^6^ 22Rv1 cells [passage, 20–25]) in 100 µL of phosphate-buffered saline (PBS) and Matrigel matrix (Corning) (1:1). The experiments began once tumors reached a volume of approximately 300 mm^3^ (4–8 wk). To reduce the levels of circulating testosterone and dihydrotestosterone, orchiectomy was performed on some of the mice after the tumors reached the desired volume. Imaging or biodistribution studies were performed 4 d after surgical castration.

### Synthesis and Characterization of F-SARM3 and B(OH)_2_-SARM3

Nonradioactive F-SARM3 and the radiolabeling precursor for [^18^F]F-SARM3, B(OH)_2_-SARM3, were synthesized via 1-step amine alkylation reactions using commercially available reagents. The synthesis and purification of both compounds are detailed in the supplemental materials. The final products were characterized with high-resolution mass spectrometry and nuclear MR using proton, ^13^C, and ^19^F probes.

### Radiosynthesis and Characterization of [^18^F]F-SARM3

A previously reported nucleophilic copper-catalyzed radiofluorination approach (12) was applied for [^18^F]F-SARM3 synthesis. The synthesis and chemical characterization of [^18^F]F-SARM3 are described in the supplemental materials.

### In Vitro Stability of [^18^F]F-SARM3 in PBS and Mouse Blood

[^18^F]F-SARM3 (185 kBq; 2–5 µL) in PBS (1.2 mL) or in athymic mouse blood (0.3 mL; drawn 5 min before the study) was incubated at 37 °C and mixed at 300 rpm in an Eppendorf Thermomixer. For PBS samples, an aliquot (0.1 mL) was used for stability analysis using radio–high-performance liquid chromatography at 0, 60, and 120 min after incubation began. For the mouse blood samples, an aliquot (0.1 mL) was centrifuged (845 × g) for 15 min to remove the blood cells. The plasma supernatant was collected and mixed with acetonitrile (0.15 mL) and placed on ice for 2 min before sample centrifugation for 5 min (9390 × g). The supernatant was collected and analyzed by radio–high-performance liquid chromatography to assess stability. Mouse blood was analyzed 15 and 120 min after incubation began.

### Human Serum Protein–Bound [^18^F]F-SARM3

A human serum sample (1.2 mL) was mixed with [^18^F]F-SARM3 (185 kBq; 3 µL in ethanol) and incubated at 37 °C. At 20 and 90 min after the start of incubation, an aliquot (0.15 mL) was mixed with acetonitrile (0.15 mL) and placed on ice for 5 min, followed by sample centrifugation for 5 min (9390 × g). The supernatant was discarded, and the protein pellet was measured using a γ-counter, along with a separate sample representing the total added activity, to calculate the fraction of activity bound to the human serum proteins.

### In Vitro [^18^F]F-SARM3 and [^18^F]FDHT Cell-Binding Assays

[^18^F]F-SARM3 (37 kBq; 2–4 µL) in PBS was added to 6-well plates seeded with LNCaP, 22Rv1, MyC-CaP, or PC3 cells (0.3–1.0 × 10^6^ cells/well), which were incubated in charcoal-stripped fetal bovine serum medium 24 h before the addition of tracer. The passage number used for the assays was less than 10 for all cell lines, but a higher passage of 27 was also used for 22Rv1 cells. For simple cell-binding assays, blocking studies were performed by coincubating treated cells with F-SARM3 or dihydrotestosterone (100 nM). For competitive cell-binding assays, the tracer was coincubated with varying amounts of dihydrotestosterone or F-SARM3 (0.01–1000 nM). The agents were incubated with the cells for 60 min at 37 °C in a 5% CO_2_ environment. The medium was aspirated, and the cells were washed twice with PBS (1 mL), followed by the addition of trypsin (1 mL) for cell collection. Cell-associated activity was measured using a γ-counter (Wizard2; Perkin Elmer). For the simple cell-binding assays, [^18^F]F-SARM3 uptake of each cell line was normalized to the average number of cells in each sample. The average number of cells was measured with a cell counter (Beckman Coulter) using separate triplicate wells that were treated identically to the other samples (vehicle plus washes with PBS).

### Measurement of F-SARM3 Half-Maximal Effective Concentration (EC_50_)

LNCaP-AR-FLUC cells were seeded in a poly-D-lysine–coated 96-well plate (5 × 10^4^ cells/well). The cells were incubated in charcoal-stripped medium for 24 h before incubating the cells in dihydrotestosterone or F-SARM3 at varying concentrations (0.01–1000 nM) for 4 h. The cells were treated with a ONE-Glo EX Luciferase Assay System (Promega Corp.) before the plate was read for bioluminescence with a SpectraMax iD5 multimode microplate reader (Molecular Devices).

### [^18^F]F-SARM3 and [^18^F]FDHT in Healthy Mice

[^18^F]F-SARM3 (6.6–6.9 MBq in 200 µL saline) or [^18^F]FDHT (8.1–8.4 MBq in 200 µL saline) was intravenously administered to healthy male nude mice (*n* = 3/cohort). The mice were imaged on a small-animal PET/CT scanner (Inveon; Siemens Healthineers) with a 10-min static scan at 1, 2, and 3 h (last time point only for [^18^F]F-SARM3) postinjection. To measure the blood pool clearance of [^18^F]F-SARM3, the half-life was evaluated in the blood of healthy male nude mice. The experimental procedure is described in the supplemental materials.

### In Vivo Study of [^18^F]F-SARM3 and [^18^F]FDHT in LNCaP Tumor–Bearing Mice

Castrated and noncastrated cohorts of LNCaP tumor–bearing male nude mice (*n* = 4–5/cohort) received intravenous [^18^F]F-SARM3 (7.0–7.4 MBq in 200 µL of saline) or [^18^F]FDHT (5.1–6.5 MBq in 200 µL of saline) via the tail vein. Two of the castrated mouse cohorts also received nonradioactive F-SARM3 (135 nmol; 30 µg; ∼1.5 mg/kg; ∼135-fold higher than the tracer mass dose). Castrated mice injected with [^18^F]F-SARM3 were imaged 60 and 120 min postinjection on a small-animal PET/CT scanner with a 10-min static scan. Two hours postinjection, all cohorts were euthanized for terminal in vivo biodistribution analysis, and all relevant organs were collected in preweighed tubes and counted with a γ-counter. For each tissue, the weight-normalized percent injected dose was calculated. Tumor tissues were collected and fixed by immersion in freshly prepared 4% paraformaldehyde and later in 70% ethanol. Later, immunohistochemistry (anti-AR antibody ER179(2)) and hematoxylin and eosin staining were performed on the sectioned tumors to study relative AR expression in noncastrated and castrated mice.

### In Vivo Study of [^18^F]F-SARM3 in 22Rv1 Tumor–Bearing Castrated Mice

Two castrated cohorts of 22Rv1 xenograft–bearing male nude mice (*n* = 4/cohort) received intravenous [^18^F]F-SARM3 (6.8–8.3 MBq in 200 µL of saline) via the tail vein. The blocking cohort also received an intravenous high mass dose of F-SARM3 (135 nmol; 30 µg; ∼1.5 mg/kg). The mice were euthanized 2 h postinjection, and in vivo biodistribution analysis was performed as described above.

### Data Analysis

All data were presented as the mean ± SD of at least 3 test replicates under identical conditions. Statistical analysis was performed using an unpaired *t*-test with Welch correction. A *P* value of 0.05 or less was considered statistically significant. Half-maximal inhibitory concentration (IC_50_) and EC_50_ curves were generated from cell uptake data using nonlinear regression with a 1-site logIC50 fit model. All data analysis was performed using Prism 10 (GraphPad Software) and ImageJ.

## RESULTS

### Development of [^18^F]F-SARM3

**FIGURE 1.**
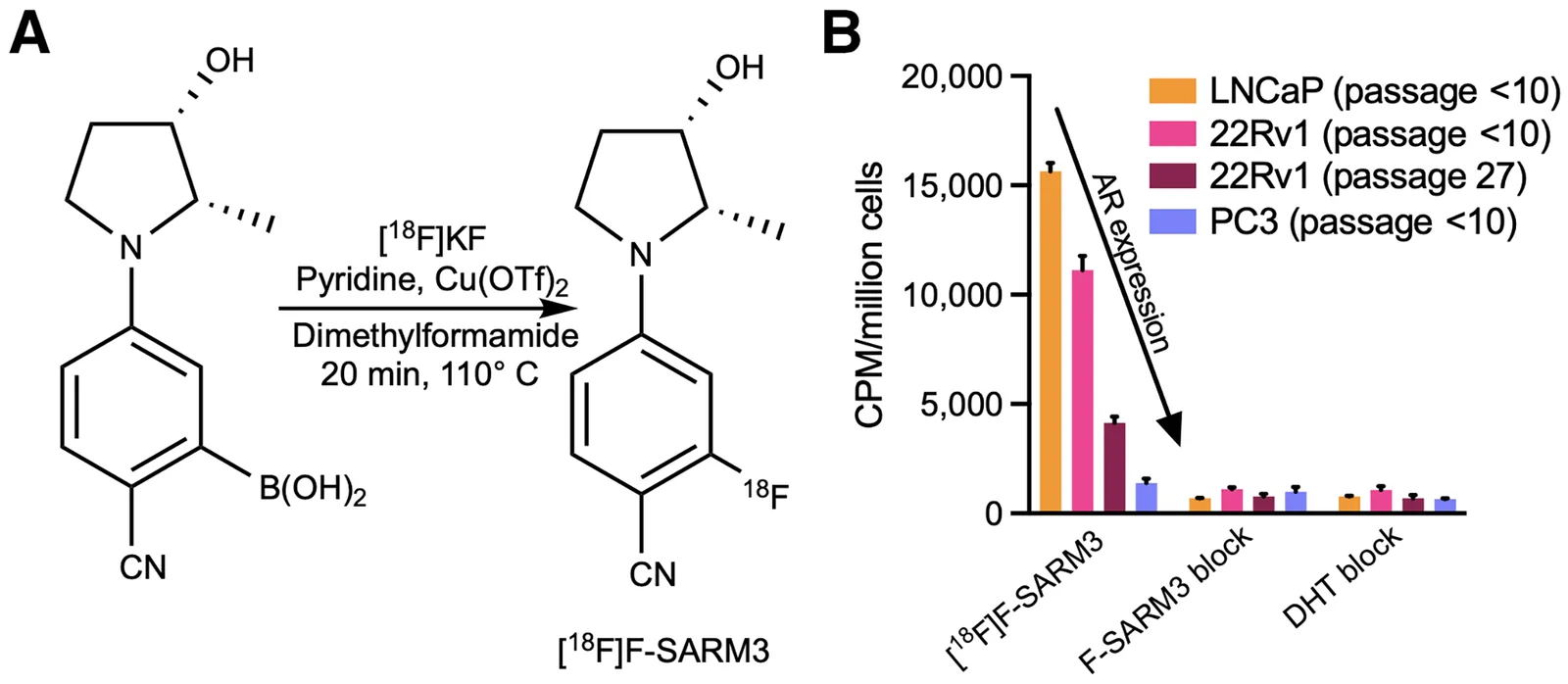
(A) Scheme for radiosynthesis of [^18^F]F-SARM3 and (B) in vitro cell-binding assay in 3 prostate cancer cell lines, revealing AR-expression–dependent [^18^F]F-SARM3 uptake that was blockable in AR-positive, LNCaP, and 22Rv1 cell lines. DHT = dihydrotestosterone.

[^18^F]F-SARM3 was synthesized with a radiochemical yield of 2.7% ± 1.4%, a radiochemical purity of 97.9 ± 2.3%, and a molar activity of 7.1 ± 4.4 GBq/µmol (*n* = 3) (Fig. 1A; Supplemental Figure 1). The radiolabeling precursor B(OH)_2_-SARM3 and the nonradioactive reference compound for the tracer (F-SARM3) were synthesized with reaction yields of 8.9% and 54.1%, respectively, and their synthesis resulted in chemical purities of 89.0% and 97.6%, respectively (Supplemental Figs. 2–4).

### In Vitro Characterization of [^18^F]F-SARM3

[^18^F]F-SARM3 demonstrated high in vitro stability in PBS and mouse blood to the last investigated time point of 120 min, where the tracer remained 98.5% ± 1.2% intact in PBS and 98.6% ± 1.5% intact in mouse blood (Supplemental Fig. 5A). Additionally, after a 90-min incubation period, a minimal amount of [^18^F]F-SARM3 was bound to human serum proteins, with 99.5% ± 1.7% of the tracer remaining unbound (Supplemental Fig. 5B).

The selectivity of [^18^F]F-SARM3 to AR was determined via cell-binding assays in MyC-CaP (high AR expression), LNCaP (moderate AR expression), 22Rv1 (moderate and low AR expression; 2 passages), and PC3 (no detectable AR expression) prostate cancer cells. [^18^F]F-SARM3 demonstrated specific binding to all AR-positive cells that were blockable with nonradioactive F-SARM3 and dihydrotestosterone (Fig. 1B; Supplemental Fig. 6) (*P* < 0.01). [^18^F]F-SARM3 showed minimal binding to PC3 cells, and its binding was not blockable with F-SARM3 (*P* = 0.07).

**FIGURE 2.**
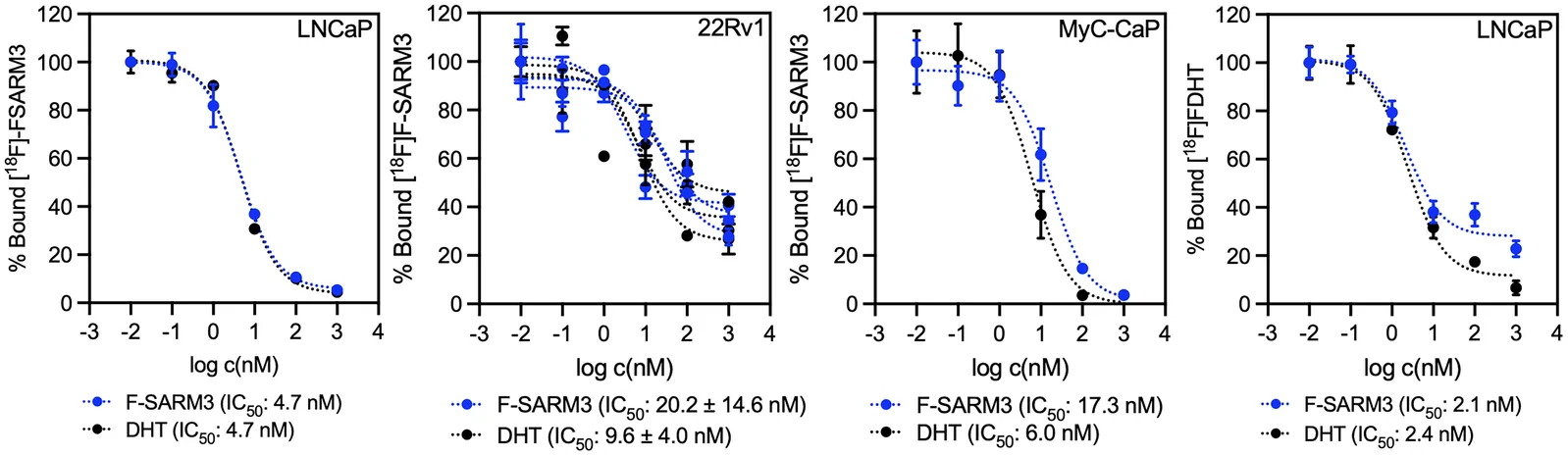
Competitive cell-binding assay demonstrated that F-SARM3 has extremely high affinity for ARs in investigated human (LNCaP and 22Rv1) and murine (MyC-CaP) prostate cancer cell lines. IC_50_ of F-SARM3 and dihydrotestosterone were similar, with all cell lines using [^18^F]F-SARM3 or [^18^F]FDHT as radioligand. DHT = dihydrotestosterone.

The in vitro affinity of F-SARM3 to ARs was quantified in LNCaP (*n* = 1), MyC-CaP (*n* = 1), and 22Rv1 (*n* = 3) cells with competitive binding assays, where [^18^F]F-SARM3 was displaced by the addition of increasing amounts of dihydrotestosterone or F-SARM3. F-SARM3 showed similar binding to ARs as did dihydrotestosterone in all cell lines (Fig. 2). The dihydrotestosterone or F-SARM3 concentrations needed to block 50% of the [^18^F]F-SARM3 binding were 4.7 nM and 4.7 nM in LNCaP cells, 6.0 nM and 17.3 nM in MyC-CaP cells, and 9.6 ± 4.0 nM and 20.2 ± 14.6 nM in 22Rv1 cells, respectively. Additionally, we studied in LNCaP cells the concentrations of dihydrotestosterone and F-SARM3 needed to block 50% of the binding of [^18^F]FDHT, which again revealed that dihydrotestosterone and F-SARM3 had similar high affinities for ARs. The IC_50_ of [^18^F]FDHT was 2.4 nM for dihydrotestosterone and 2.1 nM for F-SARM3.

**FIGURE 3.**
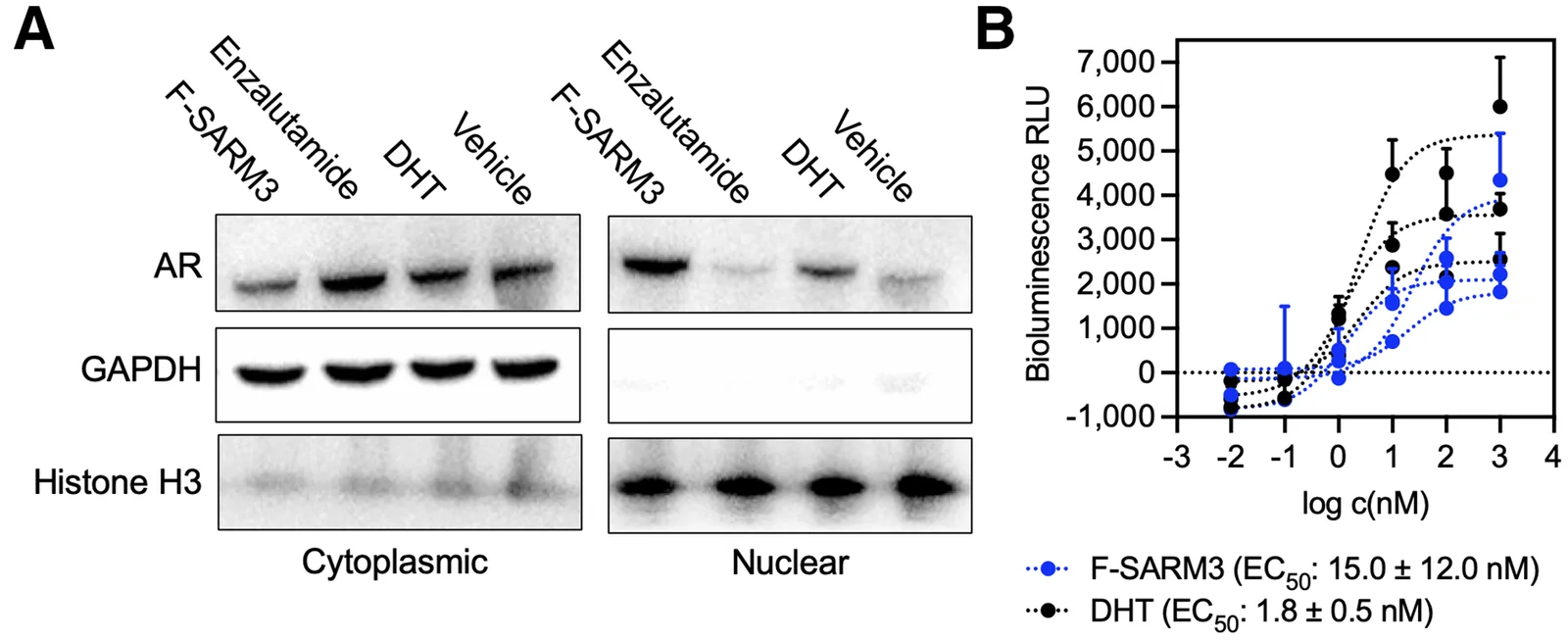
F-SARM3 is partial agonist AR ligand. (A) Cytoplasmic and nuclear AR Western blot of LNCaP prostate cancer cells treated with F-SARM3 (100 nM), enzalutamide (100 nM), and dihydrotestosterone (DHT, 100 nM) for 4 h before sample preparation. (B) Biological activity EC_50_ assay of F-SARM3 and dihydrotestosterone in LNCaP-AR-FLUC transfected cells, confirming AR activation by F-SARM3. RLU = relative light units.

To determine whether F-SARM3 was an agonistic or an antagonistic AR ligand, we performed 2 experiments. First, we quantified cytoplasmic and nuclear AR protein fractions in F-SARM3– and dihydrotestosterone-treated LNCaP cells to determine whether F-SARM3 initiates the translocalization of ARs from the cytoplasm to nucleus, similar to the full agonist dihydrotestosterone. Based on the data acquired from the nucleus/cytoplasm-isolated LNCaP Western blot samples, cells treated with F-SARM3 underwent translocalization of ARs from cytoplasm to nucleus to a higher degree compared with cells treated with the AR agonist dihydrotestosterone (Fig. 3A; Supplemental Fig. 7). The cells incubated with the AR antagonist enzalutamide or only with vehicle (negative controls) demonstrated minimal AR translocalization, as expected. The agonistic behavior of F-SARM3 was reconfirmed using AR luciferase reporter assay on a LNCaP-AR-FLUC cell line. The reporter assay revealed that F-SARM3 activated AR signaling and had a higher EC_50_ value compared with dihydrotestosterone (15.0 ± 12.0 nM vs. 1.8 ± 0.5 nM, respectively, *P* = 0.20) (Fig. 3B) and therefore can be considered a partial agonist.

### In Vivo Characterization of [^18^F]F-SARM3 and Comparison with [^18^F]FDHT

**FIGURE 4.**
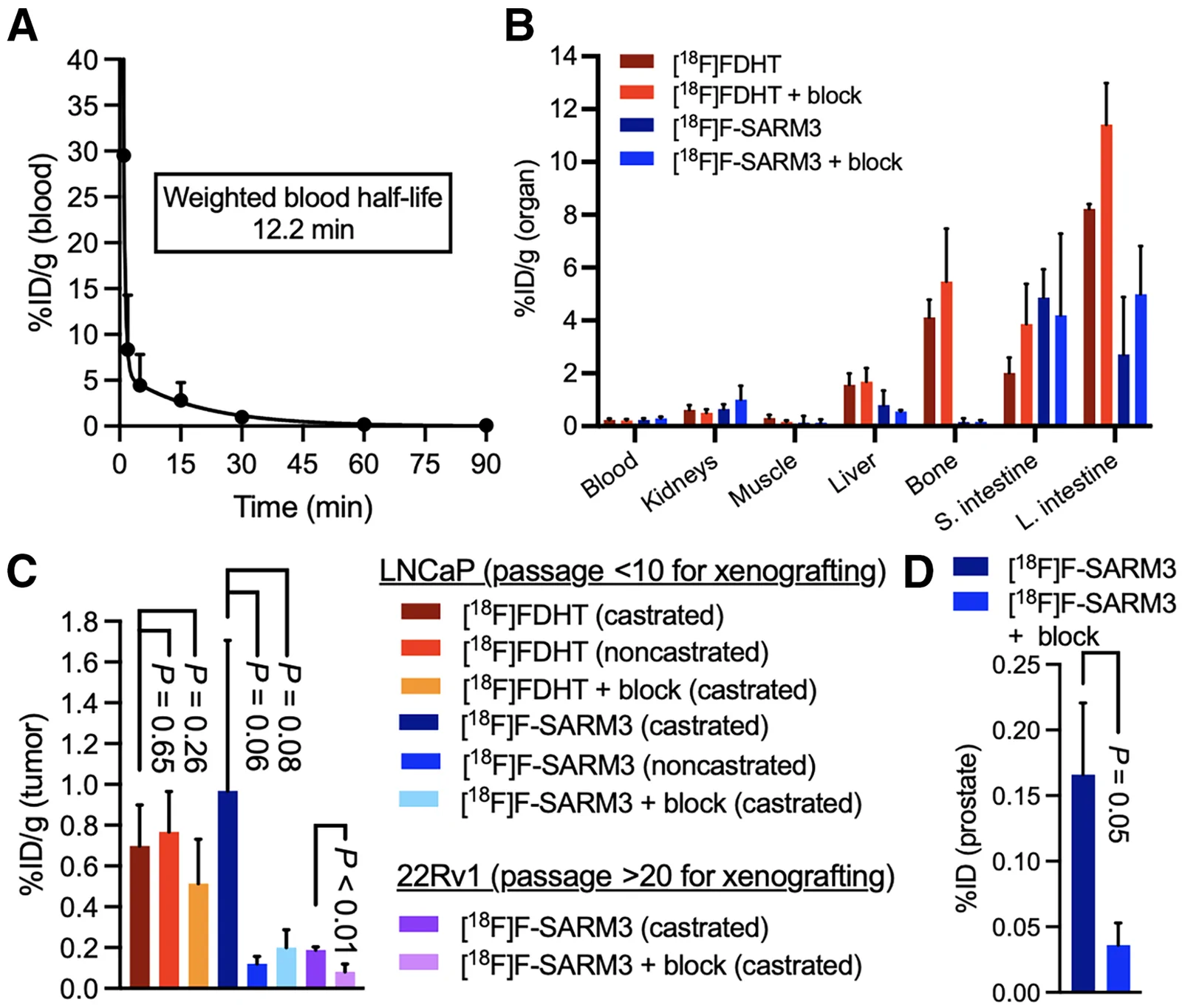
In vivo studies reveal superiority of [^18^F]F-SARM3 over [^18^F]FDHT in measuring AR status. (A) Using collected blood samples, blood time–activity curve of [^18^F]F-SARM3 in healthy male nude mice indicated rapid clearance from blood pool. (B) Extensive in vivo biodistribution studies performed in castrated LNCaP tumor–bearing mice revealed high uptake of [^18^F]FDHT in bone, indicating defluorination, whereas [^18^F]F-SARM3 displayed minimal bone uptake. (C) [^18^F]F-SARM3 uptake was blockable in both LNCaP and 22Rv1 tumor models in castrated mice, whereas [^18^F]FDHT LNCaP tumor uptake was nonspecific. (D) Specific uptake of [^18^F]F-SARM3 was observed in AR-positive prostate tissue. S. = small; L. = large

The clearance of the [^18^F]F-SARM3 from the blood pool after intravenous injection was rapid, demonstrating a biphasic clearance profile. The weighted half-life of the compound in blood was 12.2 min (Fig. 4A). The clearance of [^18^F]F-SARM3 and [^18^F]FDHT occurred via the kidneys and hepatobiliary system. On the basis of biodistribution studies at 2 h postinjection, clearance from the intestine and liver was slower with [^18^F]FDHT compared with [^18^F]F-SARM3 (liver: 1.6 ± 0.4 and 0.8 ± 0.6 %ID/g, respectively; large intestine: 8.2 ± 0.2 and 2.7 ± 2.2 %ID/g, respectively) (Fig. 4B; Supplemental Fig. 8; Supplemental Table 1). As expected, [^18^F]FDHT administration resulted in high bone uptake (4.1 ± 0.7 %ID/g) because of in vivo defluorination of [^18^F]FDHT. However, [^18^F]F-SARM3 had minimal bone uptake (0.2 ± 0.1 %ID/g), indicating that defluorination of the tracer did not occur.

In vivo biodistribution study results indicated that tumor uptake of [^18^F]F-SARM3 in castrated LNCaP tumor–bearing mice was, on average, higher in the experimental cohort than in the blocking group (1.0 ± 0.7 %ID/g and 0.2 ± 0.1 %ID/g, respectively; *P* = 0.08) (Fig. 4D). Tumor uptake of the tracer in the experimental noncastrated cohort was reduced (0.1 ± 0.0 %ID/g) compared with the castrated group, revealing that circulating dihydrotestosterone competed with the binding of the tracer to ARs (*P* = 0.06). On the basis of immunohistochemical staining of the tumors from castrated and noncastrated mice, AR expression remained unchanged after castration (Supplemental Fig. 9). However, varying degrees of necrosis were observed in tumors of both cohorts, which could potentially explain the high SD observed in [^18^F]F-SARM3 uptake of LNCaP tumors in the experimental castrated cohort. In 22Rv1 tumor–bearing mice with high passage numbers and low AR expression, we observed specific tumor uptake in castrated mice that was blockable (0.2 ± 0.0 %ID/g, *P* < 0.01) (Fig. 4C; Supplemental Fig. 10). Lower in vivo uptake in 22Rv1 tumors compared with higher uptake in LNCaP tumors correlated with the relative AR expression of these 2 models, informed by our in vitro cell-binding assays (Fig. 1B). In addition to the tumors with low AR expression, we observed specific prostate uptake of [^18^F]F-SARM3 in castrated mice (*P* = 0.045) (Fig. 4D). Unlike [^18^F]F-SARM3, the average [^18^F]FDHT uptake in tumors was similar in castrated and noncastrated experimental cohorts and in the castrated blocking cohort (0.7 ± 0.2, 0.8 ± 0.2, and 0.5 ± 0.2 %ID/g, respectively).

**FIGURE 5.**
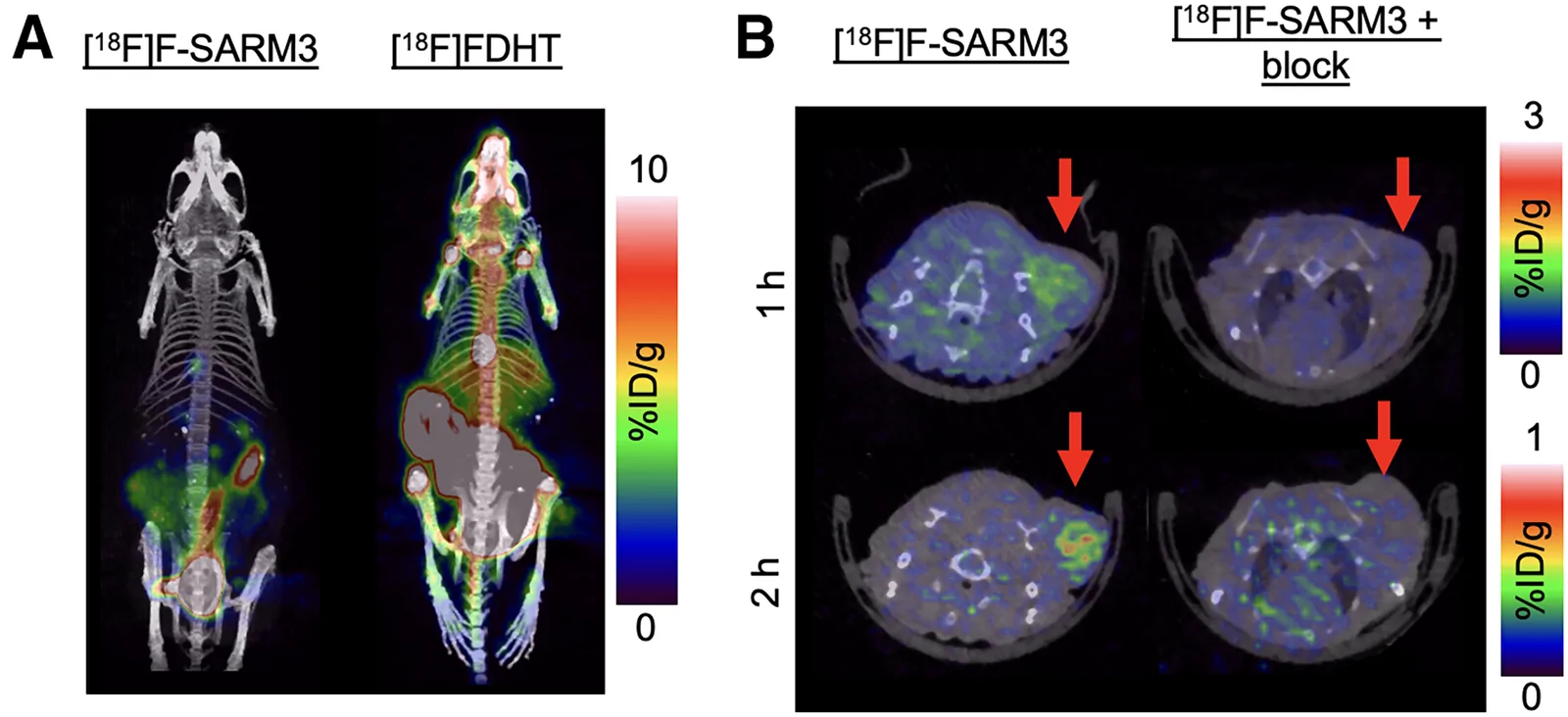
In vivo PET imaging highlighting [^18^F]F-SARM3’s improved pharmacokinetic profile compared with [^18^F]FDHT. (A) Maximum-intensity-projection [^18^F]F-SARM3 and [^18^F]FDHT PET/CT images of healthy male nude mice demonstrate [^18^F]F-SARM3’s faster clearance from nontarget tissue and lower defluorination-driven bone uptake compared with [^18^F]FDHT. (B) Axial [^18^F]F-SARM3 PET/CT images of LNCaP (red arrow) tumor–bearing castrated male nude mice, illustrating AR-positive tumor specificity of tracer (B).

Results of the PET/CT imaging study of [^18^F]F-SARM3 and [^18^F]FDHT in healthy mice agreed with biodistribution study results: [^18^F]F-SARM3 exhibited overall faster clearance and lower bone uptake because of the decreased defluorination rate detected with [^18^F]F-SARM3 compared with [^18^F]FDHT (Fig. 5A). The imaging study in castrated LNCaP tumor–bearing mice resulted in clear tumor delineation of the subcutaneous tumors at both investigated time points (60 and 120 min postinjection) (Fig. 5B; Supplemental Fig. 11). The selective blocking of the tracer with F-SARM3 was demonstrated with PET imaging, showing that tumor uptake was similar to background (muscle) in the blocking cohort.

## DISCUSSION

We reported the radiosynthesis and preclinical investigation of a nonsteroidal AR PET tracer, [^18^F]F-SARM3, that allows for the quantification and imaging of AR receptors in tumors in vivo. The only clinically studied AR tracer, [^18^F]FDHT, is not suitable to study AR occupancy in preclinical studies in rodent models because of the requisite need for [^18^F]FDHT to be bound to SHBG and rapid metabolism in rodents (4,8,9). In contrast, because of the nonsteroidal nature, high-affinity AR binding, and in vivo stability of [^18^F]F-SARM3, we observed robust uptake in vivo that correlated to AR expression. [^18^F]F-SARM3 enabled imaging of AR-positive tumors and demonstrated rapid clearance from nontarget tissues in a prostate cancer mouse model.

Over the past 2 decades, explorative human studies have been performed with [^18^F]FDHT for prostate cancer (4,6,13,14), breast cancer (15), and glioma (16). Most preclinical studies of [^18^F]FDHT have used larger animal models, such as rats, rabbits, and baboons (8,17), to study the pharmacokinetic profile, stability, and in vivo specificity of the tracer (prostate uptake) with varying SHBG presence. Previous work with [^18^F]FDHT in mouse models (no SHBG) is limited to 2 examples. Larimer et al. (9) reported the use of SHBG-bound [^18^F]FDHT to address the lack of SHBG in mouse models. They reported significantly higher [^18^F]FDHT uptake on prebound SHBG-[^18^F]FDHT administration (tumor–to–background signal) at later time points (4 h) in 22Rv1 tumor–bearing surgically castrated mice. However, administration of [^18^F]FDHT alone did not indicate specificity in either of the explored tumor models (LNCaP and 22Rv1). Further, Antunes et al. (18) compared [^18^F]FDHT with a [^18^F]F-enzalutamide AR PET tracer candidate in castrated and intact LNCaP tumor-bearing mice and concluded that [^18^F]FDHT did not have in vivo tumor specificity and the tumor–to–background signal was low (mean, ∼0.9). However, [^18^F]F-enzalutamide demonstrated specific tumor uptake, whereas the tumor–to–background signal remained low (mean, 2.2 at 1 h postinjection), unlike what we observed with [^18^F]F-SARM3 (mean, 12.2 at 2 h postinjection).

Uptake of [^18^F]F-SARM3 in healthy AR-positive tissue (muscle and prostate) was low (muscle, 0.1 ± 0.2 %ID/g; prostate, 0.2 ± 0.1 %ID) and was only blockable in the prostate (Fig. 4D). We believe that the low overall uptake in healthy tissues, especially liver and bone, observed with [^18^F]F-SARM3 will enable accurate quantification of AR expression in metastatic lesions in these organs. Compared with [^18^F]F-SARM3, we detected higher liver and bone uptake with [^18^F]FDHT (1.6 ± 0.4 %ID/g and 4.1 ± 0.7 %ID/g, respectively). Although the high bone uptake caused by defluorination of [^18^F]FDHT is reported to occur at a higher rate in mice than humans, the use of [^18^F]FDHT for the assessment of quantitative AR expression in bone metastases in humans is complicated because of the high background activity.

The radiosynthesis of [^18^F]F-SARM3 was a quick 1-step reaction with a total synthesis time of 60 min, a potential major advantage in clinical translation. The longer synthesis times (∼100 min) and 3-step synthesis required for [^18^F]FDHT increase the risk of failed synthesis and canceled or postponed clinical scans.

Because of its rapid clearance from the blood pool (Fig. 4A), [^18^F]F-SARM3 has a short accumulation time at the tumor site, resulting in relatively low uptake in tumors. It has been hypothesized that the SHBG binding of [^18^F]FDHT in humans prolongs the tracer’s half-life in blood, hence lengthening the interaction between tumor and tracer (9). We are currently investigating various approaches to increase and optimize the half-life of our tracer in blood.

## CONCLUSION

This study marked the first preclinical investigation of the nonsteroidal radiofluorinated SARM-based AR PET tracer, [^18^F]F-SARM3, in a prostate cancer mouse model. The tracer exhibited an extremely high affinity for ARs, high in vitro blood stability, in vivo specificity, and rapid clearance from nontarget tissues. [^18^F]F-SARM3 thus offers an in vivo stability and pharmacokinetic profile superior to [^18^F]FDHT, suggesting its potential for future clinical investigation.

## DISCLOSURE

Partial support was provided by NIH Cancer Center grant P30 CA08748 and NIH training grant 1T32CA254875-01 (Vilma Jallinoja) and indirectly by NIH R01 CA262675 and R01 CA274967. Vilma Jallinoja and Naga Vara Kishore Pillarsetty are the inventors of a patent describing the use of radiolabeled SARM-based agents as AR imaging agents. No other potential conflict of interest relevant to this article was reported.

## Notes

KEY POINTS **QUESTION:** Can [^18^F]F-SARM3 be harnessed to image androgen receptors in prostate cancer and how does it compare to [^18^F]FDHT? **PERTINENT FINDINGS:** Based on our findings in a prostate cancer mouse model, [^18^F]F-SARM3 uptake in vivo was specific, and [^18^F]F-SARM3 cleared from healthy tissue rapidly, unlike what was observed with [^18^F]FDHT. **IMPLICATIONS FOR PATIENT CARE:** Preclinical validation of [^18^F]F-SARM3 indicates that the tracer has significant potential to be used for monitoring AR expression in patients with prostate cancer who have undergone castration.
